# Performance Evaluation of a Novel Non-Invasive Test for the Detection of Advanced Liver Fibrosis in Metabolic Dysfunction-Associated Fatty Liver Disease

**DOI:** 10.3390/metabo14010052

**Published:** 2024-01-14

**Authors:** Anna Stefanska, Katarzyna Bergmann, Szymon Suwała, Aneta Mankowska-Cyl, Marek Kozinski, Roman Junik, Magdalena Krintus, Mauro Panteghini

**Affiliations:** 1 Department of Laboratory Medicine, Collegium Medicum in Bydgoszcz, Nicolaus Copernicus University, 87-100 Torun, Poland; bergmann@cm.umk.pl (K.B.); aneta.mankowska@cm.umk.pl (A.M.-C.); krintus@cm.umk.pl (M.K.); mauro.panteghini@cm.umk.pl (M.P.); 2 Department of Endocrinology and Diabetology, Collegium Medicum in Bydgoszcz, Nicolaus Copernicus University, 87-100 Torun, Poland; szymon.suwala@abs.umk.pl (S.S.); junik@cm.umk.pl (R.J.); 3 Department of Cardiology and Internal Diseases, Institute of Maritime and Tropical Medicine, Medical University in Gdansk, 81-519 Gdynia, Poland; marek.kozinski@gumed.edu.pl

**Keywords:** metabolic dysfunction-associated fatty liver disease, non-invasive algorithms, liver fibrosis

## Abstract

Metabolic dysfunction-associated fatty liver disease (MAFLD) may progress to advanced liver fibrosis (ALF). We evaluated the diagnostic accuracy of a novel Liver Fibrosis Risk Index (LFRI) in MAFLD subjects using transient elastography (TE) as the reference method for liver fibrosis measurement and then the diagnostic performance of a new two-step non-invasive algorithm for the detection of ALF risk in MAFLD, using Fibrosis-4 (FIB-4) followed by LFRI and comparing it to the reference algorithm based on FIB-4 and TE. We conducted a prospective study on 104 MAFLD European adult subjects. All consenting subjects underwent TE and measurements of FIB-4 and LFRI. For FIB-4 and TE, validated cut-offs were used. An ROC analysis showed that LFRI diagnosed severe fibrosis with moderate accuracy in MAFLD subjects with a negative predictive value above 90%. Using the new algorithm with LFRI thresholds recommended by the manufacturer, the number of subjects classified into ALF risk groups (low, intermediate, or high) differed significantly when compared with the reference algorithm (*p* = 0.001), with moderate agreement between them (weighted kappa (95% CI) = 0.59 (0.41–0.77)). To improve the performance of the LFRI-based algorithm, we modified cut-off points based on ROC curves obtained by dividing the study population according to the reference algorithm and observed no difference between algorithms (*p* = 0.054) in categorizing ALF risk, with a slight increase in the total agreement (weighted kappa (95% CI) = 0.63 (0.44–0.82)). Our findings suggest that using the novel LFRI as a second-line test may represent a potential alternative for liver fibrosis risk stratification in MAFLD patients; however, modified cut-offs are needed to optimize its performance.

## 1. Introduction

Metabolic dysfunction-associated fatty liver disease (MAFLD) is a new term recently introduced to highlight the importance of metabolic abnormalities as a cause of fatty liver disease, for which the prevalence in the general population is estimated at 24–36% [1,2,3]. The rising burden of MAFLD is associated with metabolic and nutritional disorders including high body mass index (BMI) and abdominal obesity, type 2 diabetes (T2D), metabolic syndrome, dyslipidemia, and long-term consumption of even moderate amounts of alcohol in combination with excessive caloric intake [1,4]. Liver fibrosis is the main factor influencing liver-related morbidity and mortality in people with MAFLD [5]. Although no universal screening methods have been proposed for MAFLD, all recent international guidelines recommend screening for advanced liver fibrosis (ALF) in suspected subjects [6,7,8]. In order to prevent MAFLD progression, the identification of patients at significant risk of ALF should therefore be easier and pursued in the primary care setting [3,5,6,7,8,9]. Although liver biopsy is still considered the “gold standard” in evaluating fibrosis severity, non-invasive methods have been proposed for detecting the presence of hepatic fibrosis in suspected MAFLD and also for identifying disease progression. Among others, transient elastography (TE), the Fibrosis-4 (FIB-4) test, and other non-invasive algorithms based on biomarkers of liver fibrosis have been proposed. TE is a non-invasive measurement system of liver stiffness associated with the degree of liver fibrosis. Although TE is a well-validated approach with a high negative predictive value (NPV) and a modest positive predictive value (PPV) for ALF, it is costly, subject to performer variability, and limited to specialized liver centers. FIB-4 is a non-invasive scoring system based on four common variables, i.e., age, serum aspartate (AST) and alanine aminotransferase (ALT) activities, and platelet count [10]. FIB-4 showed an NPV of 90% (by applying a cutoff of <1.3) and a PPV of 80% (by applying a cutoff of >3.25) for ALF [9]. The major limitation of non-invasive diagnosis of ALF is the suboptimal PPV with a grey zone representing an indeterminate diagnosis of ALF. Therefore, algorithms using a combination of non-invasive methods as second-line tests for ALF diagnosis have been proposed to increase diagnostic accuracy [11,12,13].

The Liver Fibrosis Risk Index (LFRI) is a multi-marker approach intended to provide a single score by combining the quantitative measurements of four serum markers, i.e., hyaluronic acid (HA), aminoterminal propeptide of type III procollagen (PIIINP), collagen type IV (CIV), and laminin (LN) [14,15]. These markers allow for estimating changes in the extracellular matrix molecules that comprise the extracellular scattering of the liver. The LFRI test is proposed as a second-line test for ALF risk stratification. Particularly, LFRI values < 0.028 indicate a low risk of ALF, whereas values > 0.362 are associated with a high risk of ALF, requiring patient referral to specialized centers for further management. In this study, we evaluated the diagnostic accuracy of a novel LFRI in MAFLD subjects using TE as the reference method for liver fibrosis measurement and then the diagnostic performance of a two-step non-invasive algorithm for the detection of ALF risk in MAFLD, using FIB-4 as a first line test, followed by LFRI as a second-line test, by comparing its performance to the reference algorithm based on FIB-4 and TE results.

## 2. Materials and Methods

### 2.1. Study Group

This was a prospective cohort study. The study group comprised 104 white European adult subjects (50 females (48%)) aged 21–81 years, who met the criteria for MAFLD. All subjects were recruited into this study in July 2022 and were under medical care at the Endocrinology Outpatient Clinic of the University Hospital No. 1 in Bydgoszcz, Poland. None of the subjects withdrew from participation in this study. All data were complete for each participant. The following exclusion criteria from this study were applied: history or active acute/chronic viral hepatitis, history or active autoimmune liver disease, history or active drug-induced liver injury, Wilson’s disease, hemochromatosis, history or active alcohol abuse, history or active primary sclerosing cholangitis, liver transplantation, symptomatic heart failure, history or active malignancy, treatment with drugs that may lead to drug-induced liver injury, patients with physical dysfunctions disturbing the performance of an elastography test (e.g., inability to maintain a supine position throughout the examination), and pregnancy and the breastfeeding period. MAFLD was defined as the occurrence of hepatic steatosis (S1–S3) detected using imaging techniques with the coexistence of overweight/obesity (BMI ≥ 25 kg/m^2^) or T2D or, in the case of normal weight subjects (BMI < 25 kg/m^2^), at least two of the following metabolic risk factors: waist circumference ≥ 102/88 cm in men and women, respectively, arterial blood pressure ≥ 130/85 mmHg (or specific drug treatment), triglycerides (TG) > 150 mg/dL (or specific drug treatment), HDL cholesterol (HDL-C) < 40 (females)/<50 mg/dL (males) (or specific drug treatment), prediabetes (impaired fasting glucose or impaired glucose tolerance at 2 h oral glucose tolerance test (OGTT) or glycated hemoglobin (HbA1c) 42–47 mmol/mol), homeostasis model assessment for insulin resistance (HOMA-IR) ≥ 2.5, and C-reactive protein (CRP) > 2 mg/L [16,17].

This study was conducted between June 2022 and August 2023 in accordance with the Declaration of Helsinki and approved by the Ethics Committee of Nicolaus Copernicus University in Torun, Collegium Medicum in Bydgoszcz, Poland (no. KB 828/2019 from 17 December 2019). Written consent forms were obtained from all participants before inclusion in this study. This study adhered to the Standards for Reporting of Diagnostic Accuracy Studies (STARD) guidelines [18].

### 2.2. Laboratory and Imaging Tests

Fasting venous blood samples were collected from each participant in the morning (7.00–9.00 am) into tubes containing clot activator, ethylenediaminetetraacetic acid (EDTA), and sodium fluoride. TE and blood collection were performed in accordance with international guidelines, with a particular emphasis on patient safety and identification [19,20]. In all subjects, complete blood count (CBC) was determined on an XN 1000 analyzer (Sysmex Corporation, Kobo, Hyogo, Japan). Other laboratory parameters, i.e., fasting plasma glucose (FPG), serum total cholesterol (TC), HDL-C, TG, direct LDL-cholesterol (LDL-C), total bilirubin (TB), CRP, ALT, AST, gamma-glutamyl transferase (GGT), and HbA1c, were assayed on the Alinity c platform (Abbott Laboratories, Chicago, IL, USA). Non-HDL cholesterol (non-HDL-C) was obtained by subtracting HDL-C from TC. Biomarkers of fibrosis forming the LFRI score, i.e., HA, CIV, PIIIP, and LN were measured using a chemiluminescence immunoassay on the Snibe Maglumi 800 platform (Snibe Co. Ltd., Shenzhen, China) with intra-assay precision of 2.1–6.8%, and inter-assay precision of 2.8–8.1%. The HOMA-IR value was calculated with the following formula: fasting insulin (mU/L) × FPG (mmol/L)/22.5 [21]. All laboratory tests were performed by one laboratory diagnostician at the Department of Laboratory Medicine, Collegium Medicum, Nicolaus Copernicus University in Torun, Poland. The same lot of individual reagents was used for all measurements. Hemolyzed, lipemic (TG > 1250 mg/dL), or icteric (bilirubin > 6 mg/dL) blood samples were not used.

All participants underwent TE examination for liver stiffness measurement (LSM) with simultaneous controlled attenuation parameter (CAP) determination using FibroScan^®^ Compact 530 (Echosens, Paris, France) equipped with M and XL heads, adjusted automatically depending on the clinical situation. The controlled attenuation parameter (CAP) score was used for steatosis evaluation and was graded as <248 dB/m (not significant, grade 0), 249–280 dB/m (mild, grade 1), 281–319 dB/m (moderate, grade 2), and >320 dB/m for severe steatosis (grade 3) [22,23]. The examination was performed by an experienced operator (registration: XL22900072245). During the examination, the patients lay supine with their right arm placed under their head and with a slight bend in their body to the left. LSM was performed in the projection of the right lobe of the liver through the interosseous spaces. Reliable results were those in which the ratio of the interquartile range (IQR) to the median (IQR/median LSM) did not exceed 0.3 and was optimally 0.2.

The METAVIR scoring system (Table 1) was used to assess the severity of fibrosis. The results were expressed in kPa (tissue stress/strain ratio). Then, the results were compared to the METAVIR score, relating to liver biopsy results, and assigned to an appropriate stage of hepatic fibrosis (Table 1). Liver fibrosis was defined as ≥7.9 kPa. ALF was defined as F3–F4, and results within the F2 stage (moderate fibrosis) were identified as the “grey zone” [24].

### 2.3. Non-Invasive Algorithms for Suspected Liver Fibrosis in MAFLD

The algorithms comprised a two-step non-invasive approach. The calculation of FIB-4 was first applied. Patients with FIB-4 < 1.3 (age < 65 years) or <2.0 (age ≥ 65 years) were assigned as being at low risk of ALF. Patients with FIB-4 > 3.25 were classified as having a high risk of ALF and were directly recommended for further management in a liver clinic. Patients with indeterminate FIB-4 scores (values between 1.3/2.0 and 3.25) underwent TE (reference) or LFRI (novel) testing evaluation. In this study, TE with the METAVIR score was considered as a reference method taking into account its well-established cut-offs for liver fibrosis classification.

FIB-4 and the manufacturer’s LFRI values were calculated according to the following formulas: FIB-4 = (age × AST)/(PLT × (ALT^1/2^)) [10,15]; LFRI = 0.136 ln(age) + 0.235 ln (HA) + 0.212 ln (PIIIP) + 0.165 ln (CIV) + 0.133 ln (LN) − 2.930 (equation derived from the “Model derivation and validation of multi index serological model of hepatic fibrosis”, Snibe Reagent Research & Development Center, Shenzhen, China; not published). Threshold values for FIB-4 and LFRI are reported in Table 2.

### 2.4. Statistical Analysis

Continuous variables are presented as median and IQR (25th–75th percentiles), while categorical variables are presented as numbers and percentages. The Shapiro–Wilk test was applied to test the normality of the results. The results were compared using the Fisher exact test/Chi-squared test (categorical variables) and the Mann–Whitney U-test (continuous variables). The inter-rater agreement test was used to evaluate the agreement between two algorithms with kappa values to indicate the statistical strength of concordance/discordance. Concordance was defined as an agreement between two algorithms (both algorithms give the same classification of risk), anything else was considered as discordant. The kappa value was interpreted as follows: <0.20 poor strength of agreement, 0.21–0.40 fair, 0.41–0.60 moderate, 0.61–0.80 good, and 0.81–1.00 very good. The concordance rate (%) was calculated as a ratio of the number of concordant results to all results classified as one risk group. The new LFRI threshold values were derived according to receiver operating characteristic (ROC) curves in the evaluated MAFLD subjects. In the ROC analysis, TE results with the METAVIR scoring system were used as a state variable. The area under the curve (AUC) was calculated with a 95% confidence interval (AUC, 95% CI), and thresholds with sensitivity, specificity, negative predictive value (NPV), and positive predictive value (PPV) were evaluated. During sample size determination, a significance level of 0.05 was applied for the ROC analysis with a power level of 0.9. According to the pilot study based on the first 50 subjects, we obtained AUC 0.76 for F3–F4 diagnosis with LFRI values. Based on these results, we calculated that the enrolment of 90 subjects would provide a power of 90%. The effect size (ES) was presented as Cohen’s d index (AUC values were converted to Cohen’s d index) [25]. The level of statistical significance was set as 0.05. Statistica 13.3 (StatSoft Inc., Tulsa, OK, USA) was used.

### 2.5. Cost Analysis

We estimated the direct and indirect costs related to FIB-4, TE, or LFRI testing with Polish prices (converted to Euro) for the year 2023 (Supplementary Material Table S1).

## 3. Results

The baseline characteristics of the MAFLD subjects are presented in Table 3. Overall, 60 (58%) of them had T2D, 33 (31%) were overweight (BMI ≥ 25 kg/m^2^), and 62 (60%) were obese (BMI ≥ 30 kg/m^2^).

The values of the selected biomarkers for the assessment of liver fibrosis in this study group are presented in Table 4. The LFRI values did not differ significantly between males and females (*p* = 0.97) and body mass categories (normal weight/overweight/obese: *p* for trend 0.902); however, increased significantly across quartiles of age (*p* for trend < 0.001).

**Figure 1:**
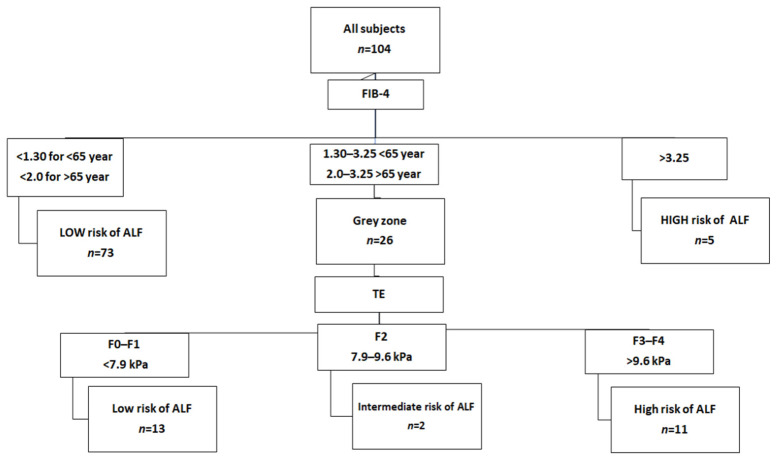
Two-step algorithm based on the combination of FIB-4 as a first-line test and TE as a second-line test for the evaluation of ALF risk in the studied subjects with MAFLD. FIB-4, Fibrosis index 4; ALF, advanced liver fibrosis; TE, transient elastography.

**Figure 2:**
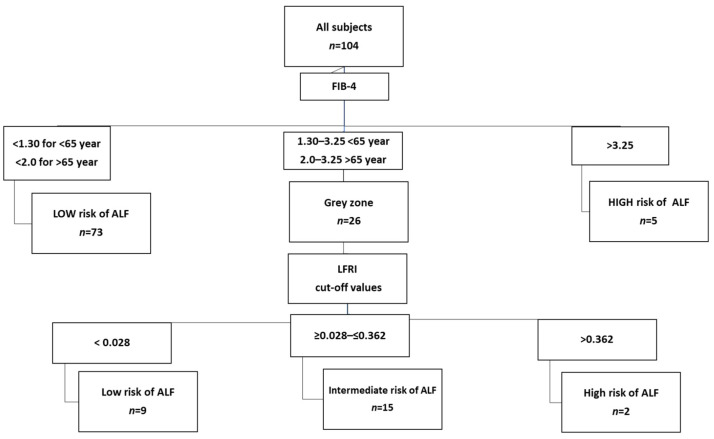
Two-step algorithm based on the combination of FIB-4 as a first-line test and LFRI as a second-line test for the evaluation of ALF risk in the studied subjects with MALFD. For LFRI, the manufacturer’s recommended thresholds were used. FIB-4, Fibrosis index 4; ALF, advanced liver fibrosis; LFRI, Liver Fibrosis Risk Index.

The risk of ALF was evaluated using a two-step algorithm in all subjects (Figure 1 and Figure 2). Initially, we assessed the performance of the reference algorithm using FIB-4 as a first-line test and TE results with the METAVIR scoring system as a second-level test. This algorithm assigned 86 (83%) subjects to the low ALF risk category, 2 (2%) subjects to the intermediate risk group, and 16 (17%) subjects to the high ALF risk group (Figure 1).

Then, we evaluated the two-step algorithm replacing the METAVIR score with LFRI using the manufacturer’s proposed thresholds. This approach assigned 82 (79%) subjects to the low ALF risk category, 15 (14%) subjects to the intermediate zone, and 7 (7%) subjects to the high ALF risk category (Figure 2).

By comparing the two algorithms, the numbers of MAFLD subjects assigned to different ALF risk groups were found to be significantly different (*p* = 0.001). An additional comparison between the two algorithms using the agreement test showed that 87 (84%) subjects were classified concordantly to ALF risk categories. Particularly, the concordance rates between the algorithms were 90% in the low, 20% in the intermediate, and 44% in the high ALF risk groups, respectively. The total agreement between the two algorithms for non-invasive ALF risk stratification was moderate (weighted kappa (95% CI) = 0.59 (0.41–0.77)).

To improve the performance of the LFRI-based algorithm, we modified its threshold points based on the ROC curves derived from our study population using the LSM/TE results classified according to the METAVIR scoring system as a reference standard. The ROC analysis showed that LFRI diagnosed F2 with poor accuracy (AUC 0.65, 95% CI 0.56–0.73) and F3-F4 with moderate accuracy (AUC 0.73, 95% CI 0.65–0.80) in the MAFLD subjects. The following newly modified thresholds were: <0.073 for F0–F1; ≥0.073–0.238 for F2; and >0.238 for F3–F4. The accuracy comparison at different cut-off points showed that the LFRI threshold values proposed by the manufacturer diagnosed the F3/F4 with markedly lower specificity (2% vs. 95%) and higher sensitivity (98% vs. 48%) when compared with the ROC-derived cut-offs. However, for both threshold values (manufacturer and ROC-derived), high NPV values were observed (Table S2 and Figure S1). The effect size (ES) of LFRI for an F2 diagnosis was medium (Cohen’s d = 0.544), while the effect size of ES for an F3-F4 diagnosis was large (Cohen’s d = 0.866).

**Figure 3:**
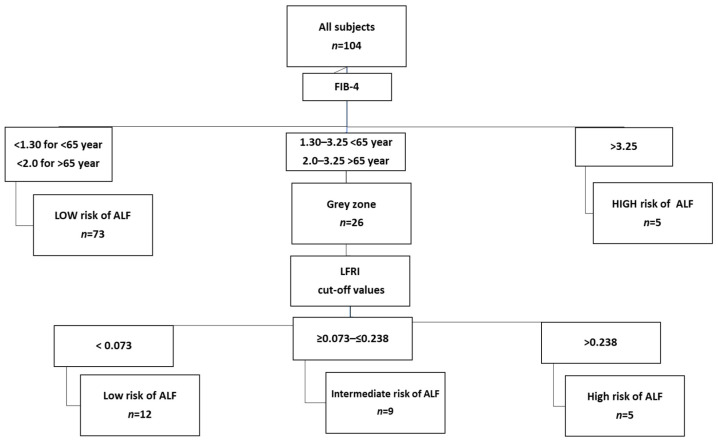
Two-step algorithm based on the combination of FIB-4 as a first-line test and LFRI as a second-line test for the evaluation of ALF risk in the studied subjects with MALFD. For LFRI, threshold values modified according to ROC curves were used. FIB-4, Fibrosis index 4; ALF, advanced liver fibrosis; LFRI, Liver Fibrosis Risk Index.

As expected, by applying these new thresholds, the evaluated FIB-4/LFRI algorithm assigned the MAFLD subjects to all risk groups similarly (*p* = 0.054) to the reference algorithm. However, while both evaluated algorithms assigned practically the same number of MAFLD individuals to the low ALF risk group ((85/104 (81.7%) vs. 86/104 (82.7%)), LFRI with modified thresholds appeared to reclassify more subjects to the intermediate and fewer to the high ALF risk group in comparison with the reference algorithm (intermediate-risk group: 9/104 (8.6%) vs. 2/104 (1.9%); high-risk group: 10/104 (9.6%) vs. 16/104 (15.3%)). The inter-rater agreement showed that 91 (87.5%) subjects were classified concordantly to the ALF risk categories. Concordance rates between the algorithms were 93%, 33%, and 56% for the low, intermediate, and high ALF risk categories, respectively. The overall agreement between algorithms for non-invasive ALF risk stratification was good (weighted kappa (95% CI) = 0.63 (0.44–0.82)) (Figure 3).

Importantly, as all individuals assigned to the intermediate and high ALF risk groups should be referred to a liver clinic, where clinicians should consider liver biopsy [12], the LFRI-based algorithm using ROC-derived thresholds showed a similar number of expected biopsies in our study population when compared to the reference TE-based one (19 vs. 18, *p* = 0.317). Higher concordance rates were observed for results indicating low ALF risk (90–93% agreement) than for those indicating the need to consider liver biopsy (63–68% agreement).

## 4. Discussion

In recent years, many non-invasive markers and algorithms for liver fibrosis risk stratification have been proposed [26,27,28,29]. Moreover, recommendations for using non-invasive tests for liver fibrosis detection in primary care have been released [30,31]. In 2022, the British Society of Gastroenterology (BSG) proposed a two-step algorithm for non-invasive diagnosis and identification of liver fibrosis in NAFLD subjects [6,13]. This approach advised simple routine liver tests (e.g., FIB-4) for initial risk evaluation, which categorizes the liver fibrosis risk in suspected individuals into three subgroups: low, intermediate, and high risk of ALF. Then, all subjects with intermediate risk, considered as a “grey zone”, should undergo second-line testing, using other non-invasive and more specific methods like TE or the Enhanced Liver Fibrosis test (ELF), which should be conducted before patients are referred to a liver clinic. Similar assumptions are presented in algorithms proposed by the American Gastroenterology Association and the American Association for the Study of Liver Disease [32,33]. Both, TE and ELF are well-established non-invasive measures for evaluating liver fibrosis risk and have been validated using liver biopsy [26,34,35]. The need to use second-line tests results from limitations of simple tests such as FIB-4. Subjects classified in the “gray zone” should be verified for a more adequate assessment of the risk of fibrosis, which may lead to a reduction in redundant biopsies and referrals to specialized hepatology clinics, as well as the organizational and financial burden on the healthcare system [36].

In this study, we evaluated the diagnostic performance of a novel non-invasive test (LFRI) based on the combination of age and four direct fibrosis biomarkers (HA, LN, PIIIP, and CIV). ELF is also based on the combination of direct markers of fibrosis, but in another combination (tissue inhibitor of metalloproteinases (TIMP-1), PIIINP, and HA) [37]. Compared to FIB-4, which is calculated based on ALT, AST, platelets, and age, the LFRI or ELF formula is based on extracellular matrix (ECM) biomarkers. An imbalance between the deposition and removal of ECM substances is associated with the development of liver fibrosis. ECM biomarkers are soluble or secreted molecules whose concentration in serum increases with hepatic fibrosis progression and decreases during effective treatment. In contrast to ELF, LFRI includes two other tests: hyaluronic acid and laminin. Although these biomarkers are not organ-specific for the liver, it has been confirmed that in patients with chronic liver diseases and fibrosis, increased production and impaired elimination of HA as well as architectural changes in the liver parenchyma leading to an increase in LN concentration are observed. Serum levels of HA and LN are correlated to the stage of hepatic fibrosis [38].

In this study, we focused on MAFLD subjects. It should be emphasized that although MAFLD patients represent a vulnerable group with higher liver stiffness and fibrosis prevalence associated with metabolic comorbidity, the recent shift from NAFLD to MAFLD has not yet been thoroughly investigated, especially in European populations [39].

In our study, the performance of LFRI was evaluated in an ROC analysis with TE as the reference method. Due to technical and financial reasons, it was not possible to perform liver biopsy as a reference method. We decided to use TE as a reference standard because of the evidence that transient hepatic elastography presents very good performance for the diagnosis and exclusion of advanced fibrosis verified by biopsy in patients with NAFLD (AUCROC > 0.9) [34,40]. We observed that LFRI diagnosed F2 (AUC = 0.65) with poor accuracy and F3-F4 with moderate accuracy (AUC = 0.73) in MAFLD subjects. We compared our results with the performance of a clinically validated indicator—ELF—because there are no available studies on the diagnostic utility of LFRI. Kjaergaard et al. observed that ELF had moderate diagnostic accuracy (AUC 0.74) for significant fibrosis assessment (TE ≥ 8 kPa) [40]. Another study showed that in patients with NAFLD, the AUC for ELF in identifying patients with advanced fibrosis was 0.81 for patients diagnosed by biopsy and 0.79 for those diagnosed by TE [41].

Secondly, LFRI was included in a two-step algorithm for the definition of ALF risk in MAFLD subjects using the classical FIB-4 approach as the first-level test. We compared this newly proposed algorithm with a well-established pathway based on FIB-4 and TE results. Being the same in the two compared algorithms, FIB-4 values ≥ 3.25 assigned only five subjects to the high ALF risk group and 26 subjects to the “grey zone”, where a second-level test is recommended [42]. It should be noted that in this study, we used the classical FIB-4 thresholds from ALF algorithms validated in NAFLD subjects, as specific cut-offs for FIB-4 in MAFLD individuals are still not available [43]. All subjects from the FIB-4 “grey zone” were reclassified according to the reference, i.e., TE results with the METAVIR scoring system, or the novel LFRI score, and their results were compared. In the LFRI scoring system, we used two classification scenarios: at first, the manufacturer’s recommended thresholds were applied. Using this approach, the LFRI results classified the studied subjects into ALF risk groups in a significantly different way in comparison with the TE results (*p* = 0.001). Therefore, we optimized LFRI with ROC-derived thresholds using the METAVIR score from our MAFLD population. This change resulted in a better performance of the newly proposed non-invasive algorithm that finally reclassified the subjects into all ALF groups similar to the TE results (*p* = 0.054). According to Kjaergaard et al., the use of two-step algorithms, particularly ELF combined with FIB-4, for liver fibrosis screening in the general population and at-risk groups reduces the number of unnecessary referrals and more cost-intensive (TE) or invasive methods (biopsy) compared with single biomarker-based tests (FIB-4 and NFS) [40].

The accuracy analysis showed that both threshold values for liver fibrosis assessment (manufacturer and ROC-derived) are characterized by high NPV values (91-98%), but lower PPV values, which was also observed by others [44,45]. We also found that the PPV values were higher for ROC-derived cut-offs and that using these threshold values gives fewer false positive results. It is known that the clinical viability of blood non-invasive diagnostic methods for liver fibrosis lies in an accurate cut-off scoring system, and this system is unclear for many proposed indexes, even those that have been known for years. Cut-off values may depend on many factors such as age, gender, ethnicity, diurnal variation, and the etiology of liver disease [46]. The weaker performance obtained when LFRI was used with the manufacturer’s recommended threshold can be possibly due to the ethnic diversity between Asian (where LFRI was implemented) and European populations such as ours (never evaluated before). The Chinese population may have a higher hereditary risk of NAFLD/MAFLD due to more frequent nonsynonymous mutations in the genes regulating lipid metabolism [47]. Moreover, the manufacturer’s proposed thresholds were obtained for the general population, while we focused on MAFLD subjects. Because the LFRI values increased significantly across quartiles of age, an evaluation of the age-dependent cut-off values for LFRI in a larger group of individuals should also be considered.

Additionally, we noted that the highest concordance rates (FIB-4/TE vs. FIB-4/LFRI) were shown for results indicating a low ALF risk, which may suggest that the second-level non-invasive tests, such as TE or LFRI, are more consistent in the low-risk assessment for excluding ALF, even in patients with MAFLD. On the contrary, the concordance rates in the intermediate- and high-risk categories were lower, which indirectly supports the recommendation that ALF risk in these groups should be verified by liver biopsy [9,45,46]. It should be mentioned that TE may have some limitations and may overestimate the risk of ALF, especially in obese individuals. With regard to this, we used an XL head in order to improve the accuracy of TE evaluation in obese subjects. Additionally, real-time elastography has been criticized for observer variability stemming [48]. It is important to emphasize that the proposed diagnostic algorithm based on biomarker testing is a non-invasive method that can be easily applied in the laboratory and does not carry any additional risk of complications or discomfort for patients. While maintaining approximately 84–87.5% of correct classifications, it is also less time-consuming and over 40% less costly than algorithms using TE (Table S1).

To our knowledge, this study is the first to address the evaluation of the LFRI score as a diagnostic tool for detecting ALF risk in MAFLD patients. Few studies have performed a comparative analysis of the available single biomarkers of fibrosis offered by Snibe on the Maglumi analyzer. A study by Stefano et al. showed that CIV concentrations > 30 µg/L were significantly associated with a greater risk of ALF in NAFLD patients [49]. Another study revealed that all mentioned biomarkers were significantly increased in patients with cirrhosis compared with controls, with HA and cholyglycine having the highest diagnostic value [50]. An unpublished study conducted at the Snibe Research & Development Center in China reported that results based on the combination of measured biomarkers showed better diagnostic accuracy than single tests [14]. Although these findings seem to be promising, further investigations in larger population-based studies are required before the LFRI score can be applied in clinical practice.

Some study limitations should be further acknowledged. We recruited a relatively low number of study participants with MAFLD for this single-center study. Additionally, we were unable to perform a liver biopsy to confirm ALF in subjects classified in both intermediate and high-risk groups using the new LFRI score. It should be also noted that the proposed threshold values are etiology-specific (MAFLD subjects) and method/manufacturer-specific (methods for serum fibrosis markers were standardized against Snibe’s internal reference standard). Larger multi-ethnic studies are needed to establish threshold values in the general population.

## 5. Conclusions

This is the first study evaluating the diagnostic performance of a novel non-invasive algorithm using the FIB-4 score as a first-line test followed by LFRI as a second-line test for liver fibrosis risk assessment. Our results seem to suggest that the LFRI score may be a potential alternative to the well-established TE method when used as a second-line test for liver fibrosis risk evaluation, especially for effectively ruling out ALF. Modified LFRI thresholds are probably needed to optimize its performance in Caucasian MAFLD patients. However, the obtained results should be considered preliminary and require confirmation in larger multicenter studies.

## Figures and Tables

**Table 1:** METAVIR scoring system for the diagnosis of liver fibrosis using elastography (FibroScan) [22].

METAVIR Score	Diagnosis	Fibroscan Cut-Off Values
F0–F1	No fibrosis to mild fibrosis—portal fibrosis without septa	<7.9 kPa
F2	Moderate fibrosis—portal fibrosis and few septa	7.9–9.6 kPa
F3–F4	Severe fibrosis—numerous septa without cirrhosis to Cirrhosis	>9.6 kPa

**Table 2:** FIB-4 and the manufacturer’s LFRI threshold values for the evaluation of liver fibrosis risk.

Index	Threshold Value	Interpretation
FIB-4	<1.30 (<65 years) <2.00 (≥65 years)	Low risk of advanced liver fibrosis
	1.30/2.00–3.25	Grey zone, perform second-line test
	>3.25	High risk of advanced liver fibrosis, refer to hepatologist
LFRI *	<0.028	Low risk of advanced liver fibrosis
	≥0.028–0.362	Moderate risk of advanced liver fibrosis
	>0.362	High risk of advanced liver fibrosis

FIB-4, Fibrosis index 4; LFRI, Liver Fibrosis Risk Index. * Threshold values established for the Asian population and proposed by the manufacturer.

**Table 3:** Baseline clinical and laboratory characteristics of the MAFLD subjects.

Variable	Subjects (*n* = 104)
Age (years)	58 (47–66)
Females (%)	48
BMI (kg/m^2^)	31 (28–34)
T2D (%)	58
FPG (mmol/L)	5.8 (5.2–7.1)
HbA1_c_ (mmol/mol)	40 (34–50)
HOMA-IR	3.2 (1.8–5.1)
TC (mg/dL)	203 (166–228)
HDL-C (mg/dL)	51 (44–61)
LDL-C (mg/dL)	118 (88–145)
TG (mg/dL)	120 (88–173)
Non-HDL-C (mg/dL)	144 (115–172)
TB (mg/dL)	0.66 (0.48–0.87)
ALT (U/L)	26 (20–40)
AST (U/L)	25 (20–34)
GGT (U/L)	26 (19–43)
CRP (mg/L)	2.2 (1.0–3.9)

Continuous variables are presented as median and IQR (25th–75th percentiles); categorical variables are presented as percentages. ALT, alanine transaminase; AST, aspartate aminotransferase; CRP, C-reactive protein; FPG, fasting plasma glucose; GGT, gamma-glutamyl transferase; HbA1c, glycated hemoglobin; HDL-C, high-density lipoprotein cholesterol; HOMA-IR, homeostatic model assessment for insulin resistance; LDL-C, low-density lipoprotein cholesterol; MAFLD, metabolic dysfunction-associated fatty liver disease; non-HDL-C, non-high-density lipoprotein cholesterol; TB, total bilirubin; TC, total cholesterol; TG, triglycerides; T2D, type 2 diabetes.

**Table 4:** Values of the evaluated biomarkers of liver fibrosis in the studied MAFLD subjects.

Variable	All Subjects *n* = 104
FIB-4	1.09 (0.77–1.48)
LSM/TE (kPa)	5.4 (4.2–8.1)
CAP/TE (dB/m)	292 (260–325)
HA (μg/L)	58 (52–65)
PIIIP (μg/L)	18 (14–28)
CIV (μg/L)	14 (12–18)
LN (μg/L)	20 (16–24)
LFRI	0.011 (−0.069–0.163)

Variables are presented as median and IQR (25th–75th percentiles); CAP/TE, controlled attenuation parameter measurement using transient elastography; CIV, type IV collagen; FIB-4, Fibrosis index 4; HA, hyaluronic acid; LFRI, Liver Fibrosis Risk Index; LN, laminin; LSM/TE, liver stiffness measurement using transient elastography; MAFLD, metabolic dysfunction-associated fatty liver disease; PIIIP, type III procollagen N peptide.

## Data Availability

The data can be made available upon reasonable request—please contact the correspondence author. The data are not publicly available due to the fact that they contain information that could compromise the privacy of research participants.

## Supplementary Materials

The following supporting information can be downloaded at: https://www.mdpi.com/article/10.3390/metabo14010052/s1, Figure S1: Diagnostic accuracy of LFRI; Table S1: Cost comparison table; Table S2: Performance of LFRI with TE as reference method.
