# Supplementary material for: Performance Evaluation of a Novel Non-Invasive Test for the Detection of Advanced Liver Fibrosis in Metabolic Dysfunction-Associated Fatty Liver Disease
Source: Metabolites. 2024 Jan 14;14(1):52. doi: 10.3390/metabo14010052 (PMC10819013; doi:10.3390/metabo14010052)
Supplement: Supplementary file 1 [file metabolites-14-00052-s001.zip › metabolites-2802011-supplementary.pdf]

Supplementary Materials

Performance Evaluation of a Novel Non-Invasive Test for the Detection of Advanced Liver Fibrosis in Metabolic Dysfunction-Associated Fatty Liver Disease

Anna Stefanska, Katarzyna Bergmann, Szymon Suwała, Aneta Mankowska-Cyl, Marek Kozinski, Roman Junik, Magdalena Krintus and Mauro Panteghini

Table S1 : Cost comparison table

Total costs per test	
FIB-4	6 euros
LFRI test	36 euros
FibroScan test	79 euros
Scenario A: FIB-4 and FibroScan as a second line test	
Cost per 100 subjects: 2654 euros	
Scenario B: FIB-4 and LFRI with the manufacturer's recommended thresholds	
Cost per 100 subjects	1536 euros
Correctly classified	84.0%
Scenario C: FIB-4 and LFRI with ROC-derived thresholds	
Cost per 100 subjects	1536 euros
Correctly classified	87.5%

FIB-4, Fibrosis-4; LFRI, Liver Fibrosis Risk Index

Table S2. Performance of LFRI with TE as reference method

LSM/TE (Metavir score)	AUC (95% CI)	Threshold value	Sensitivity (95%CI)/ Specificity (95%CI)	NPV (%)/ PPV (%)
7.9-9.6 kPa (F2)	0.65 (0.56-0.73)	≥0.073*<br>≥0.028#	63 (25-95)/72 (63-79)<br>62 (25-92)/48 (39-57)	97/12<br>95/7
>9.6 kPa (F3-F4)	0.73 (0.65-0.80)	>0.238*<br>>0.362#	48 (27-69)/95 (90-98)<br>98 (83-100)/2 (0.5-6.7)	91/65<br>98/16

*The LFRI threshold values modified according to ROC curves with the highest value of Youden index; # the LFRI threshold values established for Asian population and proposed by the manufacturer, we adapted these values to our ROC curves; NPV, negative predictive value; PPV, positive predictive value; AUC-area under the curve; LSM/TE, liver stiffness measurement using transient elastography

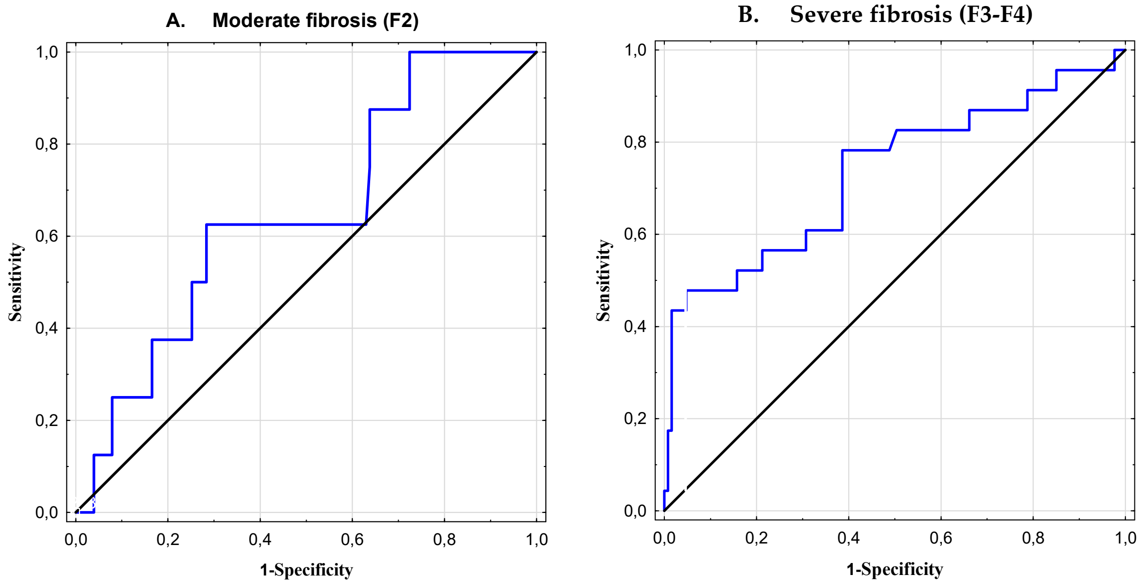

Figure S1. Diagnostic accuracy of LFRI. Area under the receiver operating characteristics curve for discriminative accuracy for liver fibrosis in MAFLD subjects with TE as reference method (A) Moderate fibrosis. (B) Severe fibrosis. LFRI, Liver Fibrosis Risk Index; TE, transient elastography
